# Leukotriene B_4_-loaded microspheres: a new therapeutic strategy to modulate cell activation

**DOI:** 10.1186/1471-2172-9-36

**Published:** 2008-07-15

**Authors:** Roberto Nicolete, Cristina Rius, Laura Piqueras, Peter J Jose, Carlos A Sorgi, Edson G Soares, Maria J Sanz, Lúcia H Faccioli

**Affiliations:** 1 Departamento de Análises Clínicas, Toxicológicas e Bromatológicas, Faculdade de Ciências Farmacêuticas de Ribeirão Preto, Universidade de São Paulo, Av. do Café s/n, 14040-903, Ribeirão Preto, São Paulo, Brasil; 2 Department of Pharmacology, Faculty of Medicine, University of Valencia, Valencia, Spain; 3 Departamento de Patologia, Faculdade de Medicina de Ribeirão Preto, Ribeirão Preto, São Paulo, Brasil; 4 Ciber CB06/06/0027 "Respiratory Diseases" Carlos III Health Institute, Spanish Ministry of Health, Madrid, Spain

## Abstract

**Background:**

Leukotriene B_4 _(LTB_4_) is a potent inflammatory mediator that also stimulates the immune response. In addition, it promotes polymorphonuclear leukocyte phagocytosis, chemotaxis, chemokinesis and modulates cytokines release. Regarding chemical instability of the leukotriene molecule, in the present study we assessed the immunomodulatory activities conferred by LTB_4 _released from microspheres (MS). A previous oil-in-water emulsion solvent extraction-evaporation method was chosen to prepare LTB_4_-loaded MS.

**Results:**

In the mice cremasteric microcirculation, intraescrotal injection of 0.1 ml of LTB_4_-loaded MS provoked significant increases in leukocyte rolling flux, adhesion and emigration besides significant decreases in the leukocyte rolling velocity. LTB_4_-loaded MS also increase peroxisome proliferator-activated receptor-α (PPARα) expression by murine peritoneal macrophages and stimulate them to generate nitrite levels. Monocyte chemoattractant protein-1 (MCP-1) and nitric oxide (NO) productions were also increased when human umbilical vein and artery endothelial cells (HUVECs and HUAECs, respectively) were stimulated with LTB_4_-loaded MS.

**Conclusion:**

LTB_4_-loaded MS preserve the biological activity of the encapsulated mediator indicating their use as a new strategy to modulate cell activation, especially in the innate immune response.

## Background

Leukotriene B_4 _(LTB_4_), a 5-lipoxygenase (5-LO)-derived eicosanoid acts as a potent chemoattractant for polymorphonuclear neutrophils (PMNs) [1], eosinophils [2] and effector T cells [3]. In addition to its direct impact on leukocyte effector functions, leukotrienes also promote innate immune responses indirectly by stimulating the production of other inflammatory mediators [4,5], inducing phagocytosis [6] and activating antimicrobial mechanisms [7,8]. Recent studies in animal models have shown that endogenous leukotrienes display protective effect against infectious diseases, including bacterial peritonitis [9], fungal pneumonia [4] and infections caused by helminths [5]. LTB_4 _binds to and activates its high-affinity receptor BLT1, which can be localized on neutrophils, eosinophils, monocytes [10] and T cells [11]. Other specific receptors for LTB_4 _are the so-called peroxisome proliferator-activated receptors (PPARs), which are situated in the cell nucleus. PPARs have been reported to regulate inflammatory responses, both *in vivo *and *in vitro*. In this context, PPARα activation by LTB_4 _binding affects the duration of the inflammatory response induced by this eicosanoid [12].

Regarding leukocyte recruitment, chemoattractants bind to their receptors on leukocytes, thereby converting rolling to firm adhesion via rapid integrin activation and/or up-regulation [13,14]. Flow cytometry studies have demonstrated that LTB_4 _triggers the up-regulation of β2-integrins on neutrophil and monocyte surface [15,16]. Several studies have suggested that lipoxygenase-triggered leukocyte adhesion might actually be due in large part to direct effects on human endothelial cells, although the relevant adhesive mechanisms have yet to be characterized [17-19]. Thus, the mechanisms by which LTB_4 _contributes to leukocyte activation and recruitment are not clearly defined.

On the other hand, previous studies have reported that LPS, TNF-α, IL-1β and LTB_4 _itself, differentially increase the expression of BLT1 and/or BLT2 receptors on HUVECs [20]. In addition, in human coronary artery, LTB_4 _also induces two important physiological responses, chemotaxis and proliferation [21]. Moreover, as demonstrated by intravital microscopy studies, LTB_4 _was very active in the microcirculation and promoted the adhesion of leukocytes to the endothelium, followed by diapedesis and migration into tissue [3]. Although it has been reported that LTB_4 _can induce CD54 (ICAM-1) expression in endothelial cells [22,23], the prevailing notion is that adhesive and migratory effects of this mediator are primarily originated from its action on the leukocyte, where chemoattractants induce up-regulation of cell-adhesion molecules that can interact with their cognate receptors on endothelial cells [24,25].

The aim of this study was to assess the activity of encapsulated LTB_4 _during *in vitro *and *in vivo *assays. In this regard, different studies have shown that systems that control antigen release can increase specific immunity by selectively driving an antigen or gene vector to immune effectors cells [26]. Based on the successful encapsulation of the LTB_4 _molecule [27] the proposed formulation can be employed on its own or combined with other therapies for the treatment of inflammatory diseases since LTB_4 _can act as immunomodulator during the inflammatory response. Other applications for this technology include the use of biodegradable polymer systems, which allow the sustained and controlled release of the encapsulated substances [28,29]. Therefore, in this study, we have evaluated the effect of LTB_4_-loaded microspheres (MS) on leukocyte-endothelial cell interactions in the murine cremasteric microcirculation. In addition, we have also investigated the effect of LTB_4 _released from microspheres in inducing MCP-1 release and nitrites production by mouse peritoneal macrophages, HUVECs and HUAECs. Moreover, in order to elucidate different mechanisms of cell activation after LTB_4_-loaded MS engulfment we assessed PPARα expression under the effect of the mediator released inside the cell, exclusively.

## Methods

### Materials

For the purposes of this study, the LTB_4 _solution, LPS of *E. coli *(serotype 0127:B8), polyvinyl alcohol, tribromoethanol and Griess reagent mixtures were obtained from Sigma Chemical Co. (St. Louis, MO, USA). Poly-lactic coglycolic acid (PLGA) polymer (50:50) was obtained from Boehringer Ingelheim (Ingelheim, Germany). Methanol, methylene dichloride, acetonitrile and acetic acid (high-performance liquid chromatography grade) were purchased from Merck (Dietikon, Switzerland). Endothelial basal medium (EBM)-2 supplemented with endothelial growth media (EGM)-2 were from Clonetics, Barcelona, Spain. The chemokine and the antibody pair for human human MCP-1 ELISA was from R&D Systems, Madrid, Spain. Neutravidin-horseradish peroxidase was from Perbio Science, Cheshire, UK. K-Blue substrate was from Neogen, Lexington, KY. The specific BLT1 receptor antagonist CP-105,696 was a kind gift from Pfizer, Inc. (Indianapolis, IN, USA).

### Ethical approval

Intravital microscopy studies with C57BL/6J mice from Charles River, weighting 23–30 g were approved by the Institutional Animal Care and Use Committee of the Faculty of Medicine, University of Valencia (Spain) and were conducted humanely.

Six- to eight-week old 5-LO^-/- ^(129-Alox5^tm1Fun^) and strain-matched wild-type (WT) male sv129 mice were obtained from Jackson Laboratories, and were bred in the Faculdade de Ciências Farmacêuticas de Ribeirão Preto, Universidade de São Paulo, Brasil. All experiments were approved and conducted in accordance with the guidelines of the Animal Care Committee of the University of São Paulo, Brazil. Mice were maintained under standard laboratory conditions.

### Preparation and characterization of the microspheres

Microspheres (MS) (diameters between 5 and 6 μm) containing LTB_4 _were prepared using an oil-in-water emulsion solvent extraction-evaporation process [27]. In brief, 0.3 ml internal organic phase (LTB_4 _3 × 10^-5 ^M, dissolved in ethanol) was added to 10 ml of methylene dichloride containing 30 mg of PLGA 50:50. This phase was poured into an external aqueous phase (40 ml of polyvinyl alcohol solution at 3% w/v) and stirred mechanically (RW20; IKA Labortechnik, Staufen, Germany) at 600 rpm for 4 h to extract the organic solvent. Finally, the microspheres formed were washed three times with doubly distilled water and then freeze-dried. All the assays were conducted with previously characterized microspheres.

### Administration of the microspheres in the mouse lungs

5-LO^-/- ^mice were anesthetized with 2.5% tribromoethanol and restrained on a small board. An anterior midline incision was made for trachea exposition. A 30-gauge needle attached to a tuberculin syringe was inserted into the trachea, and intratracheal (i.t.) dispersion was used to introduce 0.1 ml of PBS, LTB_4 _in solution (3 × 10^-8 ^M), unloaded and LTB_4_-loaded MS (5 × 10^-7 ^M or 160 ng/ml) into the lungs. PBS was the vehicle for all administrations.

### Histology

For representative histological sections that could show leukocyte infiltration in the lung parenchyma, lungs were removed on day 7 after the stimuli administrations. Then, the tissues were fixed in 10% formalin and embedded in paraffin blocks. Lung sections (5 μm) are representative of three experiments with 5-LO^-/- ^mice (5 animals each group) and were stained with hematoxylin and eosin (HE). Analysis of the sections was performed in a "blinded" manner.

### Intravital microscopy

The mouse cremaster preparation used in this study was similar to that described previously [30]. Mice were anesthetized by i.p. injection with a mixture of xylazine hydrochloride (10 mg/kg) and ketamine hydrochloride (200 mg/kg). A polyethylene catheter was placed in the jugular vein to permit the intravenous administration of additional anesthetic. The cremaster muscle was dissected free of tissues and exteriorized onto an optical clear viewing pedestal. The muscle was cut longitudinally with a cautery and held flat against the pedestal by attaching silk sutures to the corners of the tissue. The muscle was then perfused continuously at a rate of 1 ml/min with warmed bicarbonate-buffered saline (pH 7.4).

The cremasteric microcirculation was then observed by using an intravital microscope (Nikon Optiphot-2, SMZ1, Badhoevedorp, The Netherlands) equipped with a 20× objective lens (Nikon SLDW, Badhoevedorp, The Netherlands) and a 10× eyepiece. A video camera (Sony SSC-C350P, Koeln, Germany) mounted on the microscope projected the image onto a color monitor and the images were video recorded for playback analysis. Single unbranched cremasteric venules (20–40 μm in diameter) were selected for study and the diameter was measured on-line by using a video caliper (Microcirculation Research Institute, Texas A&M University, College Station, Texas). Centerline red blood cell velocity (V_rbc_) was also measured on-line by using an optical Doppler velocimeter (Microcirculation Research Institute, Texas A&M University, College Station, Texas). Venular blood flow was calculated from the product of mean red blood cell velocity (V_mean _= V_rbc_/1.6) and cross sectional area, assuming cylindrical geometry. Venular wall shear rate (γ) was calculated based on the Newtonian definition: γ = 8 × (V_mean_/D_v_) s^-1^, in which D_v _is venular diameter [31].

The number of rolling, adherent and emigrated leukocytes was determined off-line during playback of videotaped images. Rolling leukocytes were defined as those white blood cells moving at a velocity less than that of erythrocytes in the same vessel. Leukocyte rolling velocity (V_wbc_) was determined from the time required for a leukocyte to move along 100 μm length of the microvessel and is expressed as μm/s. Flux of rolling leukocytes was measured as those cells that could be seen moving past a defined reference point in the vessel. The same reference point was used throughout the experiment because leukocytes may roll for only a section of the vessel before rejoining the blood flow or becoming firmly adherent. A leukocyte was defined as adherent to venular endothelium if it was stationary for at least 30 s. Leukocyte adhesion was expressed as the number per 100 μm length of venule. Leukocyte emigration was expressed as the number of white blood cells per microscopic field surrounding the venule.

### Experimental protocol

All preparations were left to stabilize for 30 minutes. Animals were injected locally by s.c. injection beneath the scrotal skin using a 30-gauge needle with 0.1 ml (each testicle) of sterile saline, LPS (0.05 μg/kg), unloaded or LTB_4_-loaded microspheres (1 mg/ml, containing equivalent to 50 ng/ml of the solution form) and LTB_4 _in solution (200 ng/ml). Preliminary experiments indicated that local administration of 0.05 μg/kg LPS was optimal for examination of leukocyte-endothelial interaction [32]. Animals were returned to their cages for 3.5 h and the right cremaster muscle was then prepared for intravital microscopy. After 4 h of the intraescrotal injection of the agents under investigation, measurements of leukocyte rolling flux, velocity, adhesion, emigration, V_rbc,_, shear rate and diameter were obtained and recorded for 5 min.

### Cell isolation and culture

Human umbilical vein and artery endothelial cells (HUVECs and HUAECs, respectively) were isolated by collagenase treatment [33] and maintained in human endothelial cell-specific EBM-2 supplemented with EGM-2 and 10% fetal calf serum (FCS). Cells up to passage 2 were grown to confluence on 24-well culture plates. Before every experiment, cells were incubated for 16 hours in medium containing 1% FCS and then returned to the 10% FCS medium for all experimental incubations. Samples of LTB_4 _in solution (200 ng/ml) and unloaded or LTB_4_-loaded microspheres (1 mg/ml, containing equivalent to 50 ng/ml of the solution form) were added to wells. At the end of an incubation time of 4 h, cell-free supernatants were collected and stored at -20°C for MCP-1 ELISA and NO measurement by Griess reaction.

Mice peritoneal macrophages were harvested from killed mice (sv129 mice) by lavage of their peritoneal cavity with 3 ml of RPMI-1640 medium. The isolated cells were centrifuged at 400 g for 10 min and re-suspended to 3 × 10^6 ^cells/ml. Aliquots (0.5 ml) of cell suspension were added to the wells of a 24-well plate and placed overnight in a humidified atmosphere (37°C, 5% CO2) for cell adhesion. Non-adherent cells were removed by washing with RPMI-1640 medium with 10% of fetal bovine serum (FBS) and gentamycin (1 μl/ml). Firmly adhering cells (5 × 10^6 ^cells/well) were incubated for 4 h with LTB_4 _in solution (5 × 10^-8 ^M) in the absence or presence of CP 105,696 (5 × 10^-8 ^M), a specific BLT1 receptor antagonist. Similarly, cells were incubated with unloaded or LTB_4_-loaded MS (1 mg/ml, containing equivalent to 50 ng/ml of the solution form) in the absence or presence of CP 105,696. At the end of the incubation period, cell-free supernatants were collected and stored at -20°C for NO measurement by Griess reaction.

### PPAR-alpha expression assay

After 4 h incubation with the stimuli described above, peritoneal macrophages (5 × 10^6 ^cells/well) were homogenized in lysis buffer (25 mM TrisHCl pH 7.6, 150 mM NaCl, 1% NP-40, 1% sodium deoxycholate, 0.1% SDS), which contained a protease inhibitor cocktail and 100 mM PMSF, (all from Sigma, Heidelberg, Germany). After scrapping the cells they were centrifuged for 5 min, 10000 rpm at 4°C. Protein concentration was determined by using a bicinchoninic acid (BCA) protein assay kit (Pierce). Equal amounts (30 μg/lane) of protein were subjected to SDS-PAGE (10% (w/v)) gel and separated proteins were electroblotted on polyvinylidene difluoride (PVDF) membranes (Bio-Rad Laboratories). Western analysis was performed with antibodies against PPARα (ab8934) (Abcam Cambridge Science Park, Cambridge, UK) and β-actin (Sigma Aldrich).

### Densitometry

Densitometric analyses of autoradiograms were performed [34]. For each immunoblot, the pixel density I.O.D. (integrated optical density) was determined by selecting a rectangle of identical surface for all determinations, designed to entirely cover the band of interest. Background, which was subtracted from all values, was obtained using an average value from a clear area of the autoradiogram and all pixels at a black value of 255. Results were expressed as an Index = I.O.D. of stimulated cells/I.O.D. of cells cultured in medium alone.

### NO production by HUVECs, HUAECs and murine macrophages

NO production by human endothelial cells and murine macrophages was determined by Griess reaction. HUVECs, HUAECs and peritoneal macrophages were incubated for 4 h with the described stimuli above. Supernatants (0.1 ml) were incubated with an equal volume of Griess reagent mixtures (1% sulfanilamine, 0.1% N-(1-naphtyl)-ethylendiamine dihydrochloride, 2.5% H_3_PO_4_) at room temperature for 10 min. The absorbance was measured in a microplate reader at 540 nm and concentrations calculated from a sodium nitrite standard curve. Data are presented as micromoles of NO_2_^- ^(nitrite) (mean ± the SEM).

### MCP-1 release from HUVECs and HUAECs

Human endothelial cells were cultured in 24-well culture plates and stimulated as described. After coating the 96-well plates overnight with the coating anti-MCP-1 mAb, diluted supernatant samples and standards were added in PBS/0.5% BSA/0.05% sodium azide for 2 h. Biotinylated detector antibodies were added for 2 h, followed by neutravidin-horseradish peroxidase for 1 h. All plate washes were of four cycles in freshly made PBS/0.2% Tween 20. Enhanced K-Blue TMB substrate was added for 30 min and the enzyme reaction stopped by addition of 0.19 M sulphuric acid. Absorbance was read at 450 nm and the data processed by GraphPad Prism software. Results are expressed as pM chemokine in the supernatant. The sensitivity of the assay was > 10 pg/ml.

### Statistical analysis

The assays were analyzed using One-way analysis of variance (ANOVA) with post test (Tukey's Multiple Comparison Test). Values of *P *< 0.05 were considered statistically significant.

## Results

### Effect of the LTB_4_-loaded MS administration on leukocyte infiltration into the lungs and leukocyte-endothelial cell interactions

**Figure 1:**
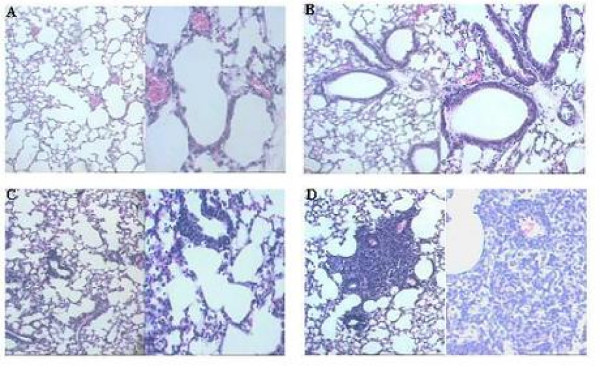
**LTB_4_-loaded MS provoke leukocyte infiltration in the lung parenchyma**. The lungs were removed on day 7 after the administrations, fixed and paraffin-embedded, followed by staining with HE. Lung sections are representative of three experiments with 5-LO^-/- ^mice (5 animals each group) that received (A) PBS; (B) LTB_4 _in solution (3 × 10^-8 ^M); (C) unloaded MS and (D) LTB_4_-loaded MS (5 × 10^-7 ^M or 160 ng/ml). Magnifications of each section: ×50 and ×400.

**Figure 2:**
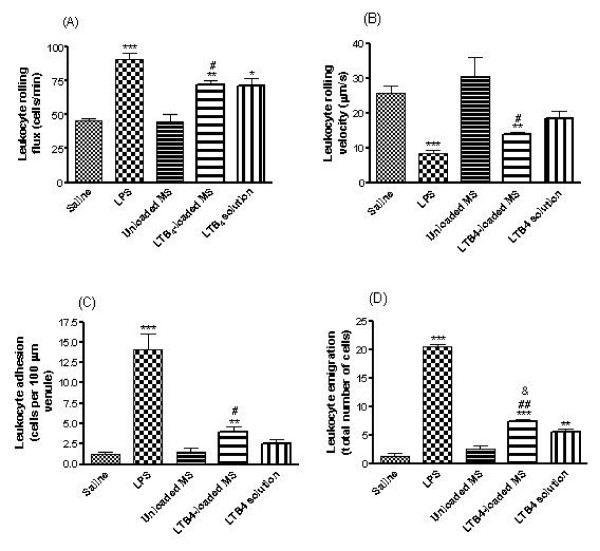
**Effect of LTB_4 _in solution and released from PLGA microspheres (LTB_4_-loaded MS) on leukocyte rolling flux (A), rolling velocity (B), adhesion (C) and emigration (D) in mice cremaster muscle postcapillary venules**. Parameters were measured 4 hours after the intraescrotal administration of saline (negative control), LPS (positive control, 0.05 μg/kg), unloaded and LTB_4_-loaded MS (1 mg/ml) and LTB_4 _in solution (200 ng/ml). Results are mean ± SEM (n = 4). *P < 0.05, **P < 0.01, ***P < 0.001, LTB_4_-loaded MS compared to saline group. # *P *< 0.05, ## *P *< 0.01 and &*P *< 0.05, LTB_4_-loaded MS compared to unloaded MS and LTB_4 _solution, respectively.

To investigate whether microspheres could release the encapsulated LTB_4 _during days, we assessed the leukocyte infiltration into the lung parenchyma of 5-LO knockout (5-LO^-/-^) mice submitted to the evaluated administrations. In this context, LTB_4_-loaded MS provoked a greater leukocyte infiltration into the lung parenchyma when compared to the other groups assayed (Fig. 1D). Intravital microscopy study was chosen to examine leukocyte-endothelial cell interactions in the mice cremasteric microcirculation. Figure 2 shows the effect of LTB_4_-loaded MS on leukocyte responses. After 4 h intraescrotal injection of 0.1 ml of 0.05 μg/kg LPS (used as control), significant increases in leukocyte rolling flux (91.0 ± 7.8 vs. 46.0 ± 1.7 cells/min), adhesion (14.0 ± 2.8 vs. 1.0 ± 1.4 cells per 100 μm vessel) and emigration (20.5 ± 0.7 vs. 1.3 ± 1.0 cells per field), and significant decreases in leukocyte rolling velocity (8.4 ± 1.9 vs. 25.9 ± 3.3 μm/s) were detected vs. values obtained in the saline treated animals. Injection of LTB_4_-loaded MS significantly increased leukocyte rolling flux (72.0 ± 5.2 vs. 46.0 ± 1.7 and 45.0 ± 7.07 cells/min), adhesion (4.5 ± 0.7 vs. 1.2 ± 0.5 and 1.5 ± 0.7 cells per 100 μm vessel) and emigration (7.3 ± 0.6 vs. 1.3 ± 1.0 and 2.5 ± 0.7 cells per field), and decreased the leukocyte rolling velocity (14.1 ± 0.9 vs. 25.9 ± 3.3 and 30.4 ± 7.9 μm/s) vs. values obtained in mice injected with saline and unloaded MS, respectively. None of these treatments had significant effects on wall shear rate (Table 1).

**Table 1:** Hemodynamic parameters in the mice cremasteric microcirculation

	Saline	LPS	Unloaded MS	LTB_4_-loaded MS	LTB_4 _solution
Venular diameter (μm)	26.5 ± 4.7	28.0 ± 3.4	19.5 ± 1.5	17.3 ± 1.2	20.0 ± 2
Venular shear rate (s^-1^)	618.7 ± 6.2	784.0 ± 1.3	843.8 ± 108.5	670.2 ± 65	879.0 ± 143.7

Parameters (mean ± SEM, in C57BL/6J mice used for intravital microscopy studies) were measured 4 h after the intraescrotal injection of saline, LPS (0.05 μg/kg), unloaded or LTB_4_-loaded MS (1 mg/ml) and LTB_4 _solution (200 ng/ml); (*n *= 4), from two different experiments.

### LTB_4_-loaded MS increase NO generation in HUVECs and HUAECs

**Figure 3:**
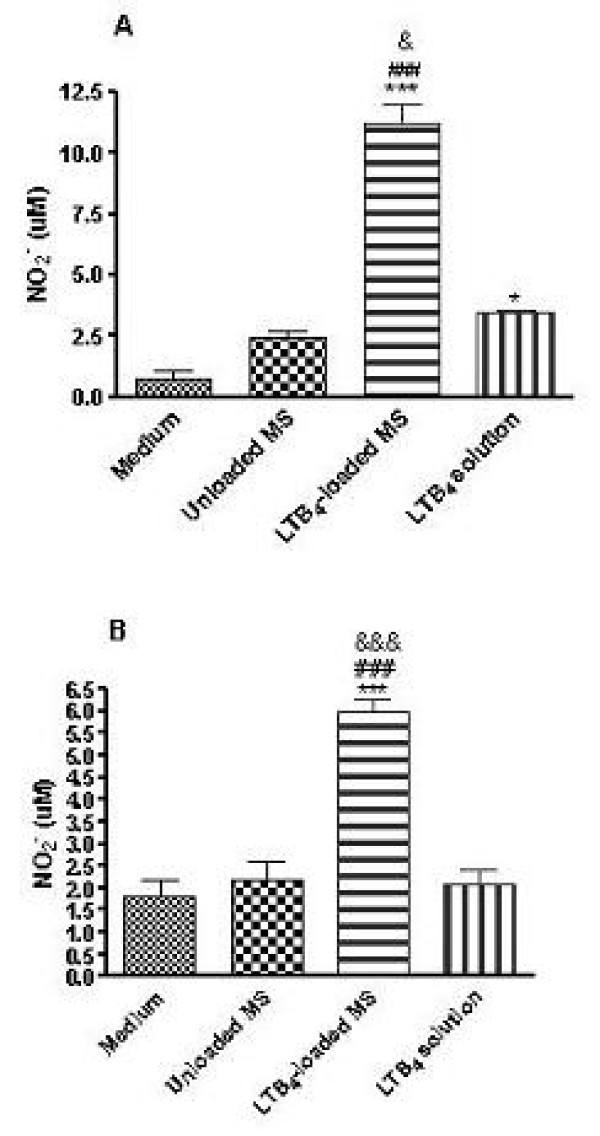
**NO production by HUVECs (A) and HUAECs (B)**. NO_2_^- ^levels were quantified by Griess reaction in the supernatants of cells incubated with medium, 1 mg of unloaded and LTB_4_-loaded MS or LTB_4 _in solution (200 ng/ml). Results are expressed as mean ± SEM (*n *= 3); *P < 0.05, ***P < 0.001, LTB_4_-loaded MS compared to control (medium). ###*P *< 0.001 and &*P *< 0.05, &&&*P *< 0.001, LTB_4_-loaded MS compared to unloaded MS and LTB_4 _solution, respectively.

Production of NO by human endothelial cells was quantified as described. In HUVECs, LTB_4_-loaded MS significantly increased nitrite levels when compared to the other stimuli (Fig. 3A). LTB_4 _in solution was also able to promote NO release although in moderate amounts. In contrast, the unloaded MS caused no significant increase in NO_2_^- ^production. On the other hand, the assay performed in HUAECs revealed that only LTB_4_-loaded MS could significantly increase the levels of nitrite (Fig. 3B).

### Engulfed LTB_4_-loaded MS increase NO generation and PPARα expression in murine macrophages

**Figure 4:**
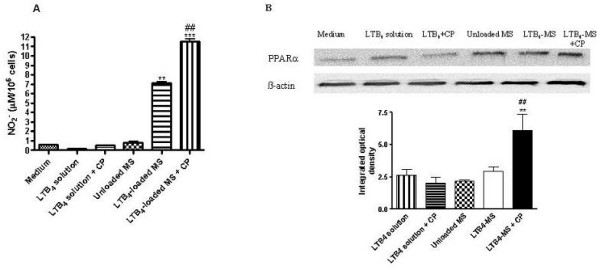
**NO production (A) and PPARα expression (B) by peritoneal macrophages after 4 hours incubation in RPMI medium**. Peritoneal macrophages were incubated for 4 h with LTB_4 _in solution (5 × 10^-8 ^M) with or without a specific BLT1 receptor antagonist (CP105,696). Similarly, cells were incubated with unloaded or LTB_4_-loaded MS (1 mg/ml) in absence or presence of CP105,696. (A) The nitrites levels were quantified by Griess reaction in the supernatants of the cells. The data are expressed as means ± the SEM (*n *= 3); **P < 0.01, ***P < 0.001, when compared to control (medium). ##*P *< 0.01, LTB_4_-loaded MS compared to LTB_4_-loaded MS + CP. (B) Western blot analysis for PPARα was performed as previously described in the Materials and Methods Section. Densitometric measurements show representative data of three separated experiments (mean ± SEM). **P < 0.01, LTB_4_-loaded MS + CP compared to LTB_4 _solution + CP. ##*P *< 0.01, LTB_4_-loaded MS + CP compared to LTB_4_-loaded MS.

We investigated nitrites levels produced by murine macrophages as a prediction of NO generation. High levels of nitrites were only achieved when the cells were stimulated with LTB_4_-loaded MS both in the presence or absence of CP 105,696 (Fig. 4A). Moreover, a further increase in nitrites production was detected when the LTB_4_-loaded MS were co-incubated with the BLT1 antagonist (CP 105,696) (Fig. 4A). Conversely, LTB_4 _in solution with or without antagonist had no effect on NO release. To assess the expression of the nuclear receptors and their possible activation under the effect of LTB_4_-loaded MS, we detected PPARα (molecular weight of approximately 52 kDa) in murine macrophages by Western Blot analysis. Treatment with LTB_4 _in solution increased PPARα expression moderately and this effect was blocked by pre-treatment of the cells with the BLT1 antagonist (CP 105,696) (Fig. 4B). However, when the cells were stimulated with LTB_4_-loaded MS, a further increase in PPARα expression was noted. Interestingly, a marked and significant increase in PPARα expression was also observed when the cells were stimulated with LTB_4_-loaded MS in the presence of the antagonist, especially when compared to that observed for the treatments with LTB_4 _solution + CP and LTB_4_-loaded MS.

### LTB_4 _released from microspheres increases MCP-1 production by human endothelial cells

**Figure 5:**
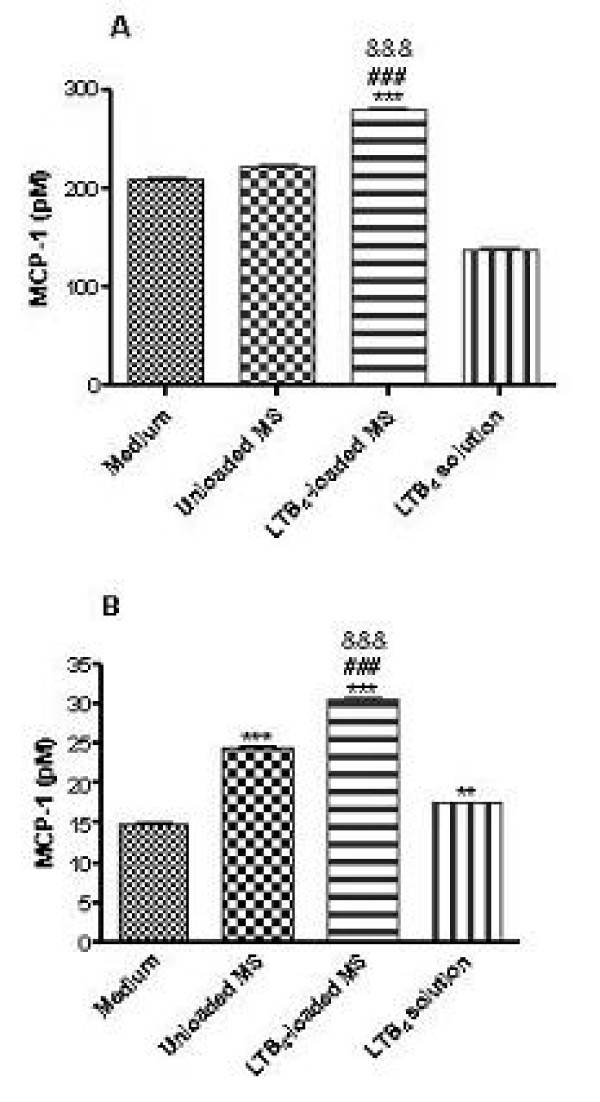
**MCP-1 levels in the supernatant of HUVECS (A) and HUAECs (B)**. Cells were incubated with 1 mg of unloaded, LTB_4_-loaded MS or LTB_4 _in solution (200 ng/ml). MCP-1 content was determined by ELISA. Results are expressed as mean ± SEM (*n *= 3); **P < 0.01, ***P < 0.001, LTB_4_-loaded MS compared to control (medium). ###*P *< 0.001 and &&&*P *< 0.001, LTB_4_-loaded MS compared to unloaded MS and LTB_4 _solution, respectively.

To evaluate LTB_4_-induced chemokine release at the cellular level, we used cultures of HUVECs and HUAECs. The amount of the chemokine released in the culture medium was determined by ELISA. In HUVECs, MCP-1 levels were significantly increased after 4 h stimulation with LTB_4_-loaded MS but not when the cells were stimulated with LTB_4 _in solution (Fig. 5A). In HUAECs, unloaded, LTB_4_-loaded MS and LTB_4 _in solution were able to increase MCP-1 contents in the supernatants investigated (Fig. 5B). Both in HUVECs and HUAECs assay, LTB_4_-loaded MS were able to increase significantly MCP-1 levels when compared to LTB_4 _solution stimulus.

## Discussion

LTB_4 _is a classical chemoattractant agent that plays a crucial role in multiple inflammatory diseases. It promotes leukocytes adhesion and diapedesis through the endothelial cell barrier. Furthermore, LTB_4 _also induces chemotaxis and proliferation of human coronary artery [21]. However, the molecular mechanisms by which LTB_4 _induces transendothelial migration of leukocytes are not completely elucidated although it seems to be involved soluble factors as well as both leukocyte and endothelial cell adhesion molecules [25]. Direct effects of LTB_4 _on endothelial cells have also been discussed, although these cells have shown a weak reaction to this mediator [35,36]. First, in this study, we investigated whether the proposed microspheres could release the encapsulated LTB_4 _during days and stimulate cells recruitment into the lung parenchyma. For this purpose, we performed an assay using 5-LO^-/- ^mice since they do not have endogenous synthesis of leukotrienes and only exogenous LTB_4 _employed could exert its biological activity. Animals that received LTB_4_-loaded MS displayed greater leukocyte infiltration in the lung parenchyma compared to PBS, LTB_4 _in solution or unloaded MS groups, demonstrating that the encapsulation method preserved the biological activity of the mediator. Although LTB_4_-loaded MS have recruited more leukocytes to the lung parenchyma than the other stimuli, the unloaded MS also provoked a marked cell infiltration. This fact can be due to the particle size (<10 μm) employed in our study since the microspheres will be preferentially engulfed by phagocytes, especially alveolar macrophages present in the lungs. The cell activation provoked by the engulfment of the microspheres suggests that other chemotactic factors or cytokines released during the particles' delivery could increase cell infiltration in the lungs. Regarding the desired pattern of cell activation, the unloaded or LTB_4_-loaded MS could be useful to stimulate the immune response against intracellular pathogens. Based on these different profiles of leukocyte infiltration provoked by the employment of the microspheres, our histological sections showed marked differences on the cell recruitment to the lungs, especially on day 7 after administrations (Fig. 1). In this regard, we previously demonstrated that the LTB_4 _released from microspheres recruited high numbers of mononuclear cells and neutrophils to the bronchoalveolar space of 5-LO^-/- ^mice [27]. Also, we conducted lung sections earlier than day 7 (days 1 and 4) but no significant leukocyte infiltration was achieved. Our findings suggest that the extended released of LTB_4 _from microspheres was able to activate and induce lung cells to generate chemotactic agents, reflected by the greater leukocyte infiltration into the parenchyma lung. These results corroborate the idea that cell migration phenomenon is strongly stimulus-dependent since exogenous LTB_4 _could prime the cells to chemotactic mediators release and recruit them to the bronchoalveolar space or lung parenchyma.

We have demonstrated that LTB_4 _released from microspheres but not LTB_4 _in solution can cause leukocyte-endothelial cell interactions within the mice cremasteric microcirculation, as demonstrated by intravital microscopy studies (Fig. 2). These results confirm that the encapsulation of the lipid mediator is an important strategy to preserve its biological activity, constituting a tool to stimulate cells. We can suggest that the microspheres might protect the LTB_4 _molecule against degradation and/or even facilitate its interaction with either the endothelium or the leukocytes.

In order to extend these findings to humans, we measured the production of nitrites and MCP-1 in response to LTB_4_-loaded MS both in HUVECs and HUAECs. In this context, different studies have shown that LTB_4 _promotes nitrites, presumably reflecting NO generation, and MCP-1 release in HUVECs and this effect is enhanced when the endothelial cells are preincubated with LPS, presumably via a functional up-regulation of BLT1 receptor [20]. In contrast, the amounts of BLT2 mRNA gradually decreased when the cells were incubated with LTB_4_. In our study, when human endothelial cells were stimulated with LTB_4_-loaded MS it was detected a massive release of nitrites and MCP-1 compared to those obtained when LTB_4 _is in solution form. This fact demonstrating the role of LTB_4_-loaded MS in increasing a chemotactic protein (as detected by MCP-1 levels) corroborates the results obtained from intravital microscopy study where we could observe more leukocytes adhered and emigrated to the cremasteric tissue after the administration of those microspheres. Therefore, our findings add further support to the hypothesis that microspheres protect LTB_4 _molecule from its metabolism, especially in this protocol in which a 4 h time period was chosen to carry out the different assays. Nevertheless, it is likely that both forms of LTB_4 _exert similar effects under acute conditions (1 h). Although unloaded MS have induced MCP-1 release by HUAECs (Fig. 5B), this fact can be due to the physical interactions between cell surface and the polymer employed to do the microspheres. Our results suggest that LTB_4_-loaded MS employed in the assays can stimulate the cells and induce a different pattern of inflammatory response compared to the mediator in solution.

In order to get more conclusive data, we also compared the effect of LTB_4 _in solution with that released from microspheres on NO production by mice peritoneal macrophages, in the presence of a specific BLT1 receptor antagonist, CP 105,696 (Fig. 4A). In this *in vitro *system, only LTB_4_-loaded MS caused a significant nitrites release whereas LTB_4 _in solution did not display any effect. Interestingly, when BLT1 receptors were blocked with CP 105,696 an enhanced and significant response was observed. This fact can be due to the different mechanisms of cell activation conferred by LTB_4 _in solution or released from MS. It is also likely that LTB_4 _released from engulfed MS could exert its activity inside the cell nucleus, where it can bind to specific receptors, whereas exogenous LTB_4 _in solution binds only to specific BLTs membrane receptors. In this context, it is known that LTB_4 _is a natural ligand for PPARα, a subtype of a family of PPARs. These transcription factors regulate gene expression of enzymes associated with lipid homeostasis and affect the duration of an inflammatory response induced by LTB_4 _[12]. Although the majority of studies have indicated an anti-inflammatory role of PPARα ligands, an increase in the neutrophil chemoattractant IL-8 and the MCP-1 levels have also been observed in endothelial cells [37]. In addition, the PPARα ligand fenofibrate was demonstrated to enhance nitric oxide synthase (NOS) expression and activity in isolated endothelial cells [38]. Also, it is important to note that some studies report that PPARγ has no anti-inflammatory activity or might indeed exert a proinflammatory response [39]. With this regard, as proposed in this study, the LTB_4 _binding to nuclear receptors without previous interaction with its membrane receptors (BLTs) results in an increase of PPARα expression (Fig. 4B) and possibly activates the inflammatory genes to produce NO, MCP-1 and other inflammatory mediators. A recent published work by our group regarding a phagocytosis' assay of LTB_4_-loaded MS [27] showed that encapsulated LTB_4 _was able to increase the number of mice peritoneal macrophages that contain microspheres and also the amount of them that were engulfed. These previous findings involving cell activation by the uptake of LTB_4_-loaded MS support our recent results showing that macrophages activation induced by LTB_4_-loaded MS can be more persistent than that with the mediator in solution.

## Conclusion

As reported in this study, the employment of LTB_4_-loaded MS seems to preserve the biological activity of the inflammatory lipid mediator as demonstrated by intravital microscopy studies as well as by *in vitro *assays using murine peritoneal macrophages and human endothelial cells. Therefore, these findings may contribute to the better understanding of the different pattern of cell activation induced by the delivery of the LTB_4 _to cell cytoplasm or nucleus and maybe these microspheres can constitute an alternative therapy for the control of different infectious diseases.

## Authors' contributions

RN designed all the experiments, carried out intravital microscopy studies, *in vitro *cell assays and drafted the manuscript. CR provided help in isolating human endothelial cells. CR and LP carried out the immunoblot assays. PJJ participated in the design of some experiments. CAS provided help in some experimental protocols. EGS carried out the histology sections. MJS and LHF participated in the design and coordination of the study and helped to draft the manuscript. All authors read and approved the final manuscript.
